# One species, two developmental modes: a case of geographic poecilogony in marine gastropods

**DOI:** 10.1186/s12862-020-01644-1

**Published:** 2020-06-26

**Authors:** Benedikt Wiggering, Marco T. Neiber, Katharina Gebauer, Matthias Glaubrecht

**Affiliations:** Department of Animal Diversity, Center of Natural History (CeNak), Universität Hamburg, Martin-Luther-King-Platz 3, 20146 Hamburg, Germany

**Keywords:** Reproductive biology, Poecilogony, Cryptic species, Larval development, Viviparity, Planaxidae, Gastropoda

## Abstract

**Background:**

Poecilogony, the presence of two developmental modes in the same animal species, is a rare phenomenon. Few cases of poecilogony have been suggested for marine invertebrates including molluscs and even less stood extensive testing, mostly revealing a species pair with differing developmental modes. We studied a textbook example of poecilogony in the viviparous snail *Planaxis sulcatus* (Gastropoda: Planaxidae), for the first time throughout its entire distribution range.

**Results:**

In the Western Indian Ocean and Red Sea this intertidal species is observed to have large, shelled juveniles, whereas in the Indo-West Pacific planktotrophic veliger larvae are released from a subhaemocoelic brood pouch. We uncovered a shift in developmental modes across its range: from west to east successively earlier developmental stages are released. Furthermore, genetic data based on mitochondrial DNA suggests to recognize *P. sulcatus* as a single species rather than a group of cryptic species. A reconstruction of the ancestral area of *P. sulcatus* based on molecular data outlines the Western Indian Ocean and the Indo-West Pacific as area of origin.

**Conclusion:**

The findings supporting *Planaxis sulcatus* as a single widespread species and the geographical shift from one reproductive mode to another suggest for this species to truly represent a case of geographic poecilogony, i.e. differing developmental modes between populations of the same species. Furthermore, the results of our ancestral range estimation imply the release of planktotrophic larvae as the ancestral developmental mode.

## Background

Developmental biology and diversity on a geographic scale are major fields of evolutionary biology studies as they oftentimes influence the interpretation and recognition of groups of individuals as species [1]. Thereby, cases where a polymorphic development is present, are of great interest as they influence our view on species recognition and interpretation of biological phenomena. Poecilogony, the presence of two different developmental modes with alternative larval types [2–8], is one of these phenomena. Such differing larval types are e.g. planktotrophic larvae (free floating larvae that feed on plankton) or lecithotrophic larvae (larvae that are nursed exclusively by the yolk originally contained within the egg). Some interpretations of the definition of poecilogony require phenotypic differences between larval types and the possibility to encounter singular female specimens producing both types of larvae in the same brood [5, 6]. Others (including the very first), differentiate geographic poecilogony including cases with phenotypically indifferent larvae but differing developmental modes while such differences are found among rather than within populations [2–4, 7, 8].

Only thirteen cases of poecilogony in marine organisms have been thoroughly studied and supported (seven spionid polychaetes [9, 10], five sacoglossan gastropods [11] and one littorinimorph gastropod [12]). For molluscs, a total of 42 cases have been suggested [5, 6], though only six cases are supported [11, 12]. The remaining cases of proposed poecilogony are either rejected or in need of detailed investigation.

The marine snail *Planaxis sulcatus* (Born, 1780) is a textbook example for poecilogony [13]. This viviparous intertidal species is widespread throughout the tropical Indo-West Pacific Region (IWP, geographic delimitations following [14]), with populations known from the Red Sea (RS), Western Indian Ocean (WIO) and Indo-Polynesian (IP) provinces. In this species, fertilized eggs are kept in a subhaemocoelic brood pouch (i.e. located in the neck region of the headfoot) until larvae or juveniles are released. Based on records form the Iranian Gulf and Pakistan populations, it has been suggested that *P. sulcatus* releasing juveniles are adelphophagic (offspring grow large by consuming other earlier developmental stages or other juveniles within the mother’s brood pouch), supplying nutrition eggs for juveniles within the brood pouch [15–17]. Concordantly, in these populations it was found that broods do not represent single cohorts, but rather that eggs can be added sequentially [17]. As in these populations later developmental stages (viz. juveniles) are far larger than earlier stages (viz. larvae), an adelphophagic nourishment is highly probable. However, no differences in larval morphology between veliger and juvenile releasing specimens has been recorded [15–20].

Six reports on the reproductive biology of this species have been published — all from singular, geographically scattered populations. In the Persian Gulf [15] and in Pakistan [16, 17] populations with long breeding periods releasing comparably large, shelled juveniles were found, whereas in Japan, Thailand [18], New Caledonia [19] and Northeast Australia [20] only planktotrophic veliger larvae were reported. These different developmental modes were either considered as an indication for poecilogony [13, 15, 21], or as revealing the presence of cryptic species [6]. In all populations studied, reproductive modes were the same within populations, differing only among populations. However, a thorough investigation of the reproductive modes throughout this species’ whole range is currently lacking, as is a molecular genetic study on potentially present cryptic species.

In this study we aim at evaluating the developmental modes in *P. sulcatus*, for the first time encompassing specimens from 71 populations across its entire range, in context with analysing molecular genetic data to clarify the potential existence of cryptic species. To investigate the geographic origin of this species and its plesiomorphic developmental mode, we conducted an ancestral range estimation based on the molecular data.

## Results

### Reproductive biology

**Fig. 1:**
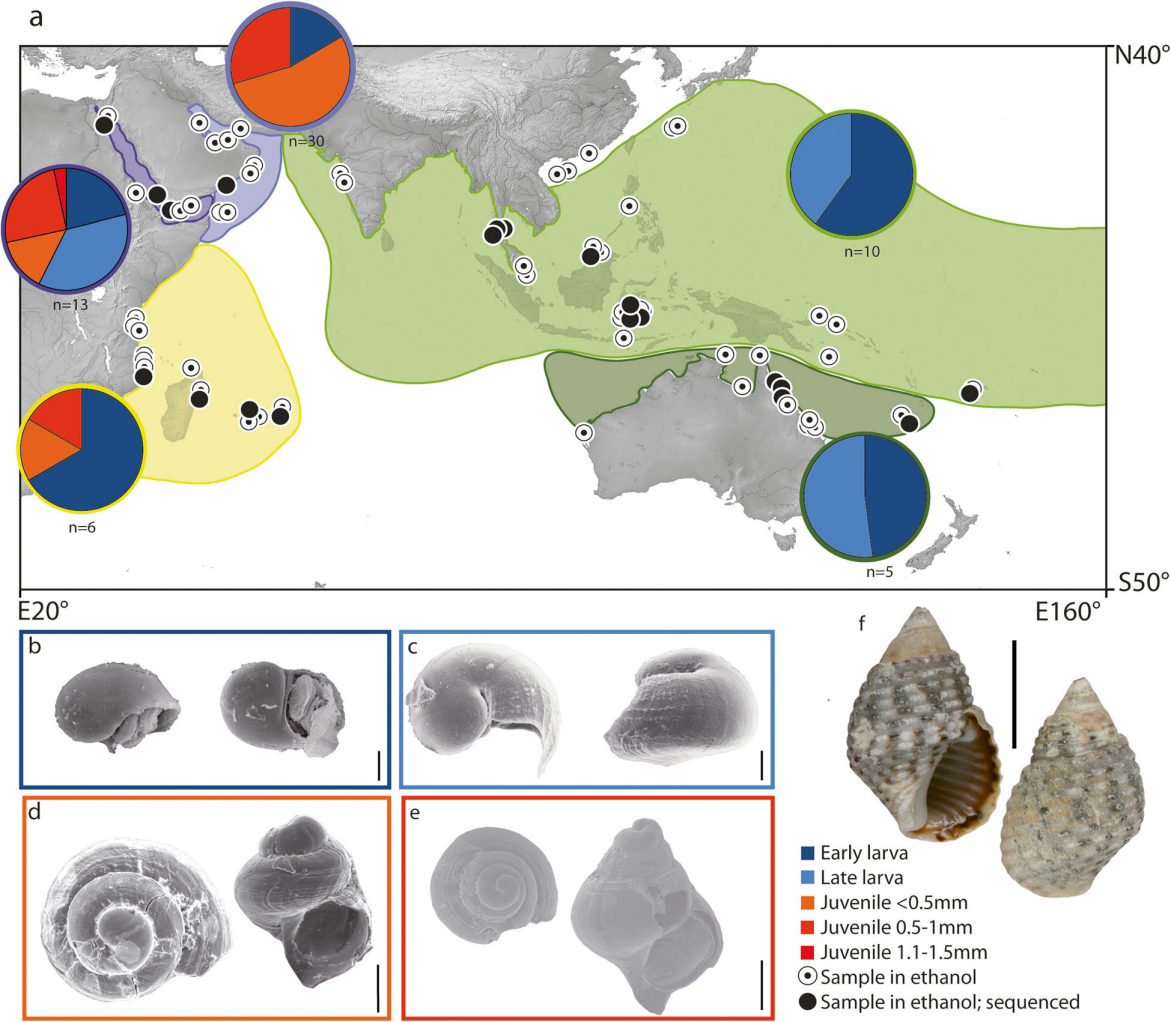
Distribution area of *Planaxis sulcatus*, (a) with brood pouch content per geographic area and (b–f) (SEM-) pictures of ontogenetic stages of *Planaxis sulcatus*. **a.** Pie charts show the cumulative proportion of each developmental stage size class in brood pouches within the five biogeographic areas, indicated by coloured shades. Numbers below each diagram indicates the number of gravid females within each region. Black dots indicate genetic samples. b–e SEM-pictures of ontogenetic stages of *Planaxis sulcatus* (Born, 1780) arranged according to size classes, in apertual and top view. **b.** Early larva, Malaysia, Borneo (ZMB 108275); **c.** Late larva, Tanzania (ZMB 108265); **d. **Juvenile < 0.5 mm, Oman, Sud (ZMB 107849); **e.** Juvenile 0.5–1 mm, Oman, Masirah (ZMB 107850); **f.** Adult, Indonesia, Southeast Sulawesi (ZMB 106003–13). Scale bars: **b, c.** = 30 μm; **d.** = 100 μm; **e.** = 200 μm; **f.** = 1 cm. Map made with Natural Earth. Free vector and raster map data @ naturalearthdata.com; for details refer to methods section

Developmental modes of *P. sulcatus* differ distinctly between geographic regions (Fig. 1). Brood pouches of IP populations contained large amounts of larvae (offspring only exhibiting a protoconch). By contrast, in RS and WIO populations, both larval stages and large juveniles (shelled offspring with a teleoconch) were present. Of the overall 364 dissected specimens 64 (17,6%) were gravid females (for a detailed list of each studied specimen and their exact brood pouch content refer to Table S3).

The number of offspring found in each brood pouch varied broadly, ranging from as few as 28 up to 10,500 individuals. Irrespective of the geographic origin, larval stages were always found in large amounts, whereas fewer individuals were present in each brood pouch when juveniles in an advanced state of development were present. In general, we found that the further developed the ontogenetic stages within a brood pouch, the fewer the amount of offspring therein.

The largest size class (juveniles 1–1.5 mm) was exclusively found within the RS populations. Regularly, all offspring within each individual brood pouch were of the same developmental stage and size class. Only 12.6% of all gravid females harboured more than one size class of offspring. In these cases, a mixture of successive size classes (for instance an admixture of 0.6–1 mm juveniles and 1.1–1.5 mm juveniles) was found.

Only within one Yemenite and one Eritrean population (both RS) one gravid female each was present containing a mixture of larval and juvenile stages within their brood pouches. The Yemenite specimen (CWR 129/84–4) contained a mixture of late larvae (*n* = 15) and small juveniles (< 0.5 mm, *n* = 80) within its brood pouch. By contrast, the Eritrean specimen (SMF 346144–4) contained a mixture of few late larvae (*n* = 35) and a relatively many large juveniles (0.6–1.5 mm, *n* = 80) within its brood pouch. Other than these two specimens, we never encountered females from any populations containing both larvae and juveniles within the same brood pouch. Furthermore, we did not find any phenotypic differences between larvae from WIO and IP populations with different reproductive modes.

### Molecular genetics

**Fig. 2:**
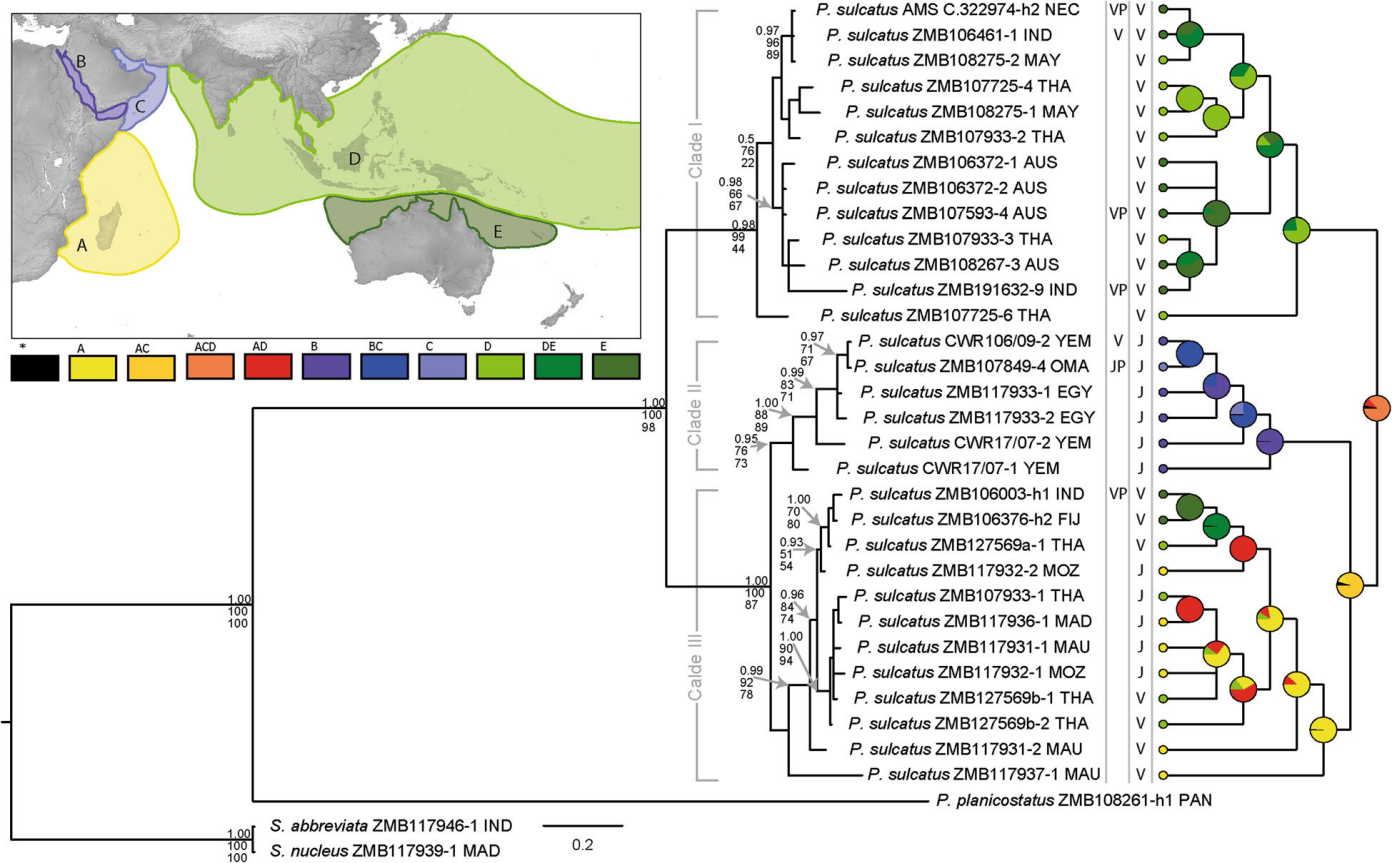
Bayesian 50% majority-rule consensus tree based on concatenated COI and 16S sequence data (left topology); nodes displaying the posterior probability (PP) values (top) from the Bayesian inference (BI), bootstrap support (BS) values from the maximum parsimony (MP) analysis (middle) and from the maximum likelihood (ML) analysis (bottom). Only nodes with PP values ≥0.95 and/or BS values from ML and/or MP analyses ≥70% are annotated. Columns to the right of the mitochondrial tree represent reproductive mode found in an individual or within the population of the sequenced individual (left column) and assumed reproductive mode based on area of origin (right column). Empty lines indicate absence of gravid females in the population. Abbreviations: V = veliger stages, VP = population found with veliger stages, J = juveniles, JP = population found with juveniles. Right topology: ancestral range estimation, with pie charts at nodes representing the estimated proportions for each area of origin. Colour codes correspond to areas depicted in the map: A = Southern West Indian Ocean, B = Red Sea, C = Northern West Indian Ocean, D = Northern Indo-West Pacific, E = Australia and New Caledonia. A combination of letters represents a shared probability of ancestral range from the corresponding regions. Abbreviations for countries of sample origin: AUS = Australia, EGY = Egypt, FIJ = Fiji, IND = Indonesia, YEM = Yemen, MAU = Mauritius, MAD = Madagascar, MAY = Malaysia, MOZ = Mozambique, NEC = New Caledonia, OMA = Oman, PAN = Panama, THA = Thailand. Map made with Natural Earth. Free vector and raster map data @ naturalearthdata.com; for details refer to methods section

Our genetic study yielded 28 16S and 16 COI sequences from a total of 32 *P. sulcatus* specimens (see Table S2 for a list of these specimens and GenBank accession numbers of individual sequences). Therefore, 19.6% of the 163 genetically sampled specimens yielded genetic data. The 32 specimens that were successfully amplified and sequenced originated from 20 populations, with eleven populations represented by one, seven populations represented by two and two populations represented by three specimens. The molecular phylogeny based on all available sequences of *P. sulcatus* revealed three distinct clades within this taxon (Fig. 2): one consisting of IP specimens (clade I), being sister to the other two, a second clade including specimens from the RS and northern WIO (clade II) and its sister clade consisting of specimens from southern WIO and IP (clade III).

The ABGD approach revealed that up to three groups can be distinguished within our COI dataset. At a K2P-value of 0.1% three groups were distinguished, aligning with clades I-III, at 1.3% two groups were found, one representing clade I and another representing clades II and III. At 3.08% all partitions collapsed (see Table S4 for more details).

The bGMYC approach based on the COI dataset suggested the same groupings as the ABGD approach (see Fig. S1 for a probability matrix based on the BEAST COI tree).

The ancestral range estimation suggests a highly probable combined IP and WIO origin (RASP Value (SV) = 86.68%) of *P. sulcatus* (Fig. 2). The different genetic clades, however, show different probabilities of origin: clade I is of mainly IP (SV = 77.35%) origin, whereas clade II is proposed to be of mainly Northern WIO origin (SV = 99.58%). Lastly, although consisting of samples from Southern IP and Southern WIO specimens, clade II is highly probable to be of combined Southern WIO and RS (SV = 86.23%) origin.

## Discussion

This study corroborates the presence of two developmental modes within *P. sulcatus*: one with large amounts of planktotrophic veliger larvae being released, and a second one with small amounts of shelled juveniles hatching from the female’s brood pouch. Contrasting prior studies that provided snapshots of distant populations [15–20], our more comprehensive study uncovered a gradually changing pattern of variation within developmental modes across the entire distribution range (Fig. 1). Generally, the further to the east and south the smaller were the juvenile shells within brood pouches with only larval stages being present in populations east of Pakistan (i.e. IP populations). Within the RS only juvenile stages were present. However, in the WIO both, juveniles and larvae, were found.

A co-occurrence of larval and juvenile stages within the same brood pouch was only observed at one Yemenite and one Eritrean locality. The Yeminite population contained one gravid female with late larvae and small juveniles (CWR 129/84–4). In this case we assume that all larvae of this specimen would have developed into juveniles, rather than being released as a mixture of veligers and juveniles as the encountered offspring was from consecutive developmental stages and class sizes. In other populations in this region, much larger juveniles were present, lending support for our assumption. The Eritrean specimen (SMF 346144–4) is an outstanding exception, as the presence of early larvae and large juveniles (0.6–1.5 mm) could potentially be interpreted as two reproductive modes being present. As specimens from both this Yemenite and the Eritrean population did not yield any molecular data, we cannot provide further insights into these critical populations. Neither our nor any other study on *P. sulcatus* indicated in any other case the presence of both reproductive modes within the same population [15–20].

Interpreting larval stages within brood pouches of conserved specimens as direct implications for present reproductive mode could, however, be ambiguous. As each specimen is sampled at a fixed point in time, there is no saying if larvae would have been released as veliger or developed further into juveniles within the brood pouch. However, the alternative – determining reproductive mode by observing the hatching/release of offspring from brood pouches directly – is logistically very challenging, due to the geographic scope of the present study. As our samples have been collected between 1921 and 2013, and throughout different seasons of these years, we regard the potential hypotheses that brood pouch content patterns are just coincidental as unlikely and, accordingly, assume the approach to infer reproductive mode by this trait as valid.

The findings of the Eritrean specimen (SMF 346144–4) containing both early larvae and large juveniles could alternatively be a hint at adelphophagy in *P. sulcatus*, as it has been suggested that larger offspring feed on developing eggs (so called nurse eggs) [15–17]. This is further hinted at by the observation that the further developed the ontogenetic stages within a brood pouch are the fewer the amount of offspring present therein. This correlation might be an indication to not only nurse eggs being consumed by developing offspring but rather all kinds of siblings might be the nourishment of the offspring. In contrast to other studies [15–17], we did not find any distinct nurse eggs. Therefore, to unambiguously solve the question of offspring nourishment in *P. sulcatus* further studies, expanding the limits of our dataset, are needed.

The genetic study reveals three clades of *P. sulcatus* (Fig. 2). The ABGD approach showed that the maximum K2P-distance within the COI gene of all specimens is 3.08%. Other marine caenogastropods show an intraspecific variance between 0.31 and 4.11%. For instance, the intraspecific variance of two similarly distributed littorinid species is 1.36 and 3.48% [22]. The bGMYC approach suggested similar groupings as the ABGD approach (Fig. S1). It must be noted that molecular species delimitation models work best for groups with a low intraspecific and high interspecific variation. In other cases, these methods usually show a tendency to oversplitting [23]. The potential intraspecific variance of 3.08% that we found for *P. sulcatus* might suggest that population structure is detected in this case rather than distinct species. Except for developmental modes, no morphological differentiation within *P. sulcatus* can be observed [see also 15, 17, 18, 20] and a geographic restriction without partial overlap is not present within the molecular clades. Furthermore, developmental modes do not entirely correlate with genetic clades. Hence, we see the genetic differences found in our study to represent intraspecific variation and reject the hypothesis [6] that *P. sulcatus* should be considered as a complex of cryptic species.

However, only 36% of the individuals, from which DNA was extracted proved to be suitable for PCR amplification. Besides the protocol listed in the material and method section [24, 25] we tried other protocols that, however, all failed to extract any additional specimens. As our study was based on museum material sampled between 1921 and 2013, with most samples collected during the 1980s and 1990s, a major hurdle of this study was finding primers and extraction protocols that actually yield DNA given this rather “old” – i.e. degraded – museum material. Consequently, an exact genetic coverage of each gravid specimen could not be realized. Hence, we interpret genetic and brood pouch content information as proxies for their respective geographic areas, rather than as direct information obtained from specific individuals in all cases.

Alternatively, the two or three genetic lineages in *P. sulcatus* could be interpreted as representing distinct species. However, this still would include at least one lineage in which the two differing developmental modes are present (see clade III in Fig. 2). Following the notion of not unnecessarily multiplying additional arguments (and taxa), we here refrain from expanding our discussion beyond the scope covered by our data, which hint at the existence of only one poecilogonous species. *Planaxis sulcatus* therefore exhibits a developmental mode, in which planktotrophic veliger larvae are released (predominant in IP populations) and another where larvae are retained for a longer period only to be released as juveniles (predominant in WIO and RS populations).

We did not find any obvious morphological differences between larval stages, excluding poecilogony in *P. sulcatus* according to the strict definition suggested by Hoagland & Robertson [5]. However, following the original definition of poecilogony [3] and more recent discussions [8], we adhere to a broader definition of the term where no morphological difference but only a polymorphism of developmental modes is the necessary criterion. Consequently, we see *P. sulcatus* to represent a case of geographic poecilogony.

Finally, the results of the ancestral range estimations imply a shared geographic origin of *Planaxis sulcatus* from IP and WIO (Fig. 2). As most other Planaxidae release veliger larvae from their brood pouches, it was assumed that this is the plesiomorphic mode of reproduction in this family [20]. Therefore, it appears likely that the spawning of pelagic veliger larvae is the ancestral developmental mode in *P. sulcatus* as well, as this is the main developmental mode of *P. sulcatus* in the ancestral regions.

Thus, we infer a scenario where from the centre of the IP adjacent areas have been colonised by ancestral lineages of this species*.* These consecutively evolved the developmental mode we now see within WIO and RS populations. Brooding larger juveniles may have ensured a higher rate of survival by sheltering the progeny from environmental conditions being different in the WIO and RS.

Prior studies suggested higher sea surface salinity [6] and intertidal zoning [17] as factors influencing the emergence of different reproductive modes. Especially changing salinities might be important as it has often been proposed that the evolution of viviparity in freshwater snails is correlated with adaptation to lower salinities [21]. However, in *P. sulcatus* it would be an increase in salinity triggering the same response. Although these factors are plausible, sea current activity might play an important role as well, as veligers are more prone to sea current drift. In a non-poeciolognous species it has been shown that depending on substrate (as an indicator for sea current activity), different life history stages hatch from laid eggs [26]. A similar factor might be at hand here, where sea currents are unfavourable for dispersal of WIO and RS populations and, hence, the longer breeding in this area evolved. In other species, poecilogony was proposed as a bet-hedging strategy, to evade unstable conditions in mudflat estuaries [27]. As *P. sulcatus* inhabits intertidal rocky shores, a similar effect might be possible.

However, to further identify the ecological drivers behind geographic poecilogony in *P. sulcatus,* controlled aquaria experiments should be conducted. Life samples from WIO, RS and IP populations could each be held under the same salinity and temperature conditions, examining at which stage each population releases its offspring, potentially revealing one of the factors as a trigger for one of the developmental modes. Unfortunately, our current dataset is not suitable to address this question. However, we anticipate that poecilogony in *P. sulcatus* is a direct result of an ecological component leading to and maintaining these differential modes of development.

## Conclusion

Our study implies the presence of poecilogony in the widespread *Planaxis sulcatus*, with a pattern of geographic variation from one developmental mode to another across its distribution range, varying in the developmental stage at which offspring are released. The ancestral range estimation shows that *P. sulcatus* originated in the WIO or IP, thereby suggesting the release of larval stages to be the initial reproductive mode. Hence, instead of assuming cryptic species our study corroborates *P. sulcatus* to truly represent a textbook example of poecilogony.

## Methods

### Material

We studied museum specimens of *P. sulcatus,* originating from a total of 71 populations from throughout the IWP, accumulated from several independent collecting trips of MG during the 1990th to early 2000 years, now stored in the Museum für Naturkunde, Berlin (ZMB, Germany), supplemented by material from several other museum collections, including ZMB, Universität Rostock (CWR, Germany), Senckenberg Naturmuseum, Frankfurt (SMF, Germany), Australian Museum, Sydney (AMS) and the Natural History Museum, London (NHMUK, United Kingdom). These samples were collected between 1921 and 2013, though most samples (42) were collected before the year 2000 (see Table S1 for a detailed list of all used samples and sample locations).

### (a) Analysis of brood pouch content for developmental mode evaluation

We studied brood pouch contents of gravid females, to identify reproductive modes within *P. sulcatus* populations. As all previous studies on the matter [15–20] never identified a co-occurrence of reproductive modes within the same population, we interpret finding only larval stages within brood pouches of a given population to represent the veliger releasing mode, whereas we regard populations containing any juvenile stages within brood pouches as indication of direct development. However, this interpretation is not unambiguous as the brood pouch contents only provide a snapshot of the developmental stages present within a population at the time of sampling. Larval stages could still develop into juveniles within the brood pouch or be released at an earlier stage. To account for this ambiguity we combined results from singular populations with other populations from the same area, to infer the reproductive biology in a given region. As our samples were collected between 1921 and 2013 it is highly unlikely that for any given region all samples were collected during the same season, reducing the possibility of a seasonal sampling bias.

In total, 365 adult specimens were dissected using a Leica M125 stereo microscope (Leica Microsystems GmbH, Wetzlar, Germany). If present, ontogenetic stages were extracted and counted according to predefined size classes: early larva, late larva, juveniles < 0.5 mm, juveniles 0.5–1 mm, juveniles 1–1.5 mm; see Fig. 2 b–e. We here define larvae as individuals with only a protoconch and juveniles as individuals with a teleoconch. This transition is easily spotted in *P. sulcatus* as the transition from teleoconch to protoconch is marked by a deep sinusigeral notch [20]. Following Nation [28], larval and juvenile stages were dried for subsequent imaging with a LEO 1525 GEMINI scanning electron microscope (SEM, LEO Electron Microscopy Inc., Thornwood, NY, USA). Dried larval and juvenile stages were mounted on SEM object stubs (Agar Scientific Ltd., Stansted, UK) and coated with platinum using a Polaron SC7640 sputter coater (Quorum Technologies Ltd., Ashford, UK).

### DNA extraction & amplification

DNA was extracted from foot muscle tissue using mollusc specific protocols [24, 25]. Wherever possible specimens with offspring of known developmental mode in their brood pouches were preferentially used. We performed extractions on 2–3 specimens per studied population, amounting to a total 163 genetically sampled specimens. We added sequences of a confamilial species to our dataset as outgroup: *Supplanaxis abbreviata* (Pease, 1865) (see Table S1 for a comprehensive list of used samples). Partial sequences of the mitochondrial cytochrome c oxidase subunit I (COI) gene, with primers LCO1490 (5′-GGT CAA CAA ATC ATA AAG ATA TTG G-3′ [29]) and HCO2198var (5′-TAW ACT TCT GGG TGK CCA AAR AAA T-3′ [30]), and 16S rRNA (16S) gene, with primers 16SF (5′-CCG CAC TAG TGA TAG CTA GTT TC-3′ [31]) and H3059var (5′-CCG GTY TGA ACT CAG ATC ATG T-3′ [31]) were amplified by polymerase chain reaction (PCR). Amplifications were performed in 20 μl volumes containing 2 μl 10x DreamTaq Green Buffer, 0.1 μl DreamTaq DNA polymerase (both Thermo Fisher Scientific, Waltham, MA, USA), 0.4 μl dNTP mix 10 mM each (VWR chemicals, VWR International GmbH, Darmstadt, Germany), 1 μl of each primer (Sigma-Aldrich Chemie GmbH, Taufkirchen, Germany), 13.5 μl ddH_2_O and 2 μl DNA template under the following reaction conditions: initial denaturation at 94 °C for 3 min, 35 PCR cycles (94 °C for 30 s, 50 °C for 45 s, 72 °C for 1 min), final extension at 72 °C for 10 min. PCR products (10 μl) were cleaned enzymatically by adding 2 μl FastAP Thermosensitive Alkaline Phosphatase (1 U/μl) and 1 μl Exonuclease I (20 U/μl) (both Thermo Fisher Scientific) followed by an incubation step at 37 °C for 15 min and inactivation at 85 °C for 15 min. All amplified products were sequenced at Macrogen Europe (Amsterdam, The Netherlands). See Table S2 for GenBank accession numbers for all obtained sequences.

### Phylogenetic analysis

DNA sequences were edited and assembled using GENEIOUS R 9.1.3 (Biomatters Ltd., Auckland, New Zealand). Primer sequences were removed resulting in COI sequences of ~ 658 bp and 16S sequences of ~ 810 bp. COI sequences were aligned using MUSCLE [32] as implemented in GENEIOUS under default settings. For the 16S alignment MAFFT 7 [33], using the Q-INS-I algorithm, the 1PAM/κ = 2 option for the scoring matrix for nucleotide sequences and otherwise default settings, was used. We used PartitionFinder 2.1.1 [34] to select the appropriate partitions and evolutionary models. Four partitions were assumed initially (1st, 2nd and 3rd codon positions of COI and 16S). The results of the PartitionFinder analysis using the Bayesian information criterion suggested a single partition and the HKY + G model, which was used for the subsequent Bayesian inference (BI) and maximum likelihood (ML).

We performed a BI using MrBayes version 3.2.6 [35] running Metropolis-coupled Monte Carlo Markov chain (MC^3^) searches with four chains in two separate runs for 50,000,000 generations with trees sampled every 1000 generations under default heating. Potential scale reduction factors close to 1 and estimated effective sample sizes above 200 from the MrBayes output were used as diagnostics to ensure that the MC^3^ searches had reached stationarity and convergence. The first 5,000,000 generations of each run were discarded as burn-in. We performed heuristic ML analyses in GARLI 2.0 [36] using the best-fit model as suggested by PartitionFinder. Support values were computed by bootstrapping with 1000 replications. Using PAUP* 4.0b10 [37], we conducted heuristic maximum parsimony (MP) searches with unordered characters, 100 random sequence addition replicates, the tree bisection and re-connection (TBR) branch-swapping, and gaps treated as missing data. Internal branch support was assessed in PAUP* by bootstrapping with 1000 replications, using full heuristic searches with 10 random addition sequence replicates, TBR branch swapping, and one tree held at each step during stepwise addition. Posterior probabilities from the BI analysis and bootstrap support (BS) values from the ML and MP analyses were mapped onto the BI 50% majority-rule consensus tree with SumTrees version 3.3.1 (part of the DendroPy 3.8.0 package [38]). BS ≥ 70% from the ML and MP analyses and posterior probabilities (PP) ≥ 0.95 were interpreted as positive support for a node.

### Molecular species delimitation models

To test for the presence of potential cryptic lineages we used the Automated Barcode Gap Discovery (ABGD method) [39], via its online application (http://wwwabi.snv.jussieu.fr/public/abgd/abgdweb.html). For the COI gene we used the Kimura (K80) TS/TV 2.0 distance (K2P). We entered previously established [22] minimum (Pmin = 0.0031) and maximum (Pmax = 0.0411) COI K2P values for marine caenogastropod snails (Littorinidae) and otherwise default settings.

We also used the general mixed Yule-coalescent (GMYC) approach in its Bayesian implementation (bGMYC) [40] for DNA sequence-based species delimitation. We constructed ultrametric trees based on COI data with BEAST 2.4.1 [41]. The chain was run for 11,000,000 generations, with a sample frequency of 1000. The first 1000,000 of the generations were discarded as burn-in. The GTR + I + G model was applied; otherwise default settings were used. Tracer 1.7.1 [42] was used to check that all effective sample sizes were above 200. GMYC and bGMYC analyses were conducted with the Split [43] and bGMYC R packages [44], respectively. The single-threshold as well as the multiple-threshold analyses were both conducted using the maximum clade credibility tree from the BEAST analysis constructed with TreeAnnotator 2.1.2 (part of the BEAST software suite) setting the posterior probability limit to 0. The bGMYC analysis was based on 100 trees drawn equidistantly from the post burn-in generations obtained from the BEAST analysis. For each of the 100 trees, the Markov-chain Monte Carlo sampler was run for 100,000 generations, discarding the first 90,000 generations as burn-in and sampling every 100 generations.

### Ancestral range estimation

An ancestral range estimation was conducted based on 1000 randomly selected post-burn-in trees from the BI analysis of the dataset accounting for statistical uncertainty and the 50% majority-rule consensus tree from the BI analysis using the statistical dispersal-vicariance analysis (S-Diva) method [45] implemented in RASP 4.0 beta [46]. We constructed a matrix in which each individual was assigned to one of five geographic regions (roughly following [14]): A) Southwest Africa, Madagascar and Mauritius; B) the Red Sea; C) the Arabian Sea (including the Gulf of Yemen); D) the Indo-West Pacific (including Indonesia, Malaysia and Thailand) and; E) Australia and New Caledonia. For our analysis, we allowed transitions from B only to A and C, as well as from E only to D. All other areas were assumed as directly connected. The analysis was run allowing a maximum of three areas at each node and otherwise default settings.

### Distribution maps and figure assembly

The species’ distribution was reconstructed on a dot by dot basis based on ethanol stored specimens from the aforementioned collections, with maps based on the open access Natural Earth map (free vector and raster map data @ naturalearthdata.com). Figures and maps were assembled using Adobe Photoshop CS2 version 9.0 for Windows and Adobe Illustrator CS2 version 12.0 for Windows (both Adobe Systems, San Jose, CA, USA).

## Supplementary information

**Additional file 1 Table S1** Material examined. **Table S2** Specimens examined for the molecular genetic analysis of this study with GenBank accession and inventory numbers. **Table S3** Results of brood pouch content analysis of *Planaxis sulcatus*. If present, content was counted according to five predefined size classes. **Table S4** Results of ABGD analysis for the COI mt rRNA dataset using the K2P model to calculate pairwise distances. P is the prior maximum intraspecific divergence. **Fig. S1** Pobability matrix of the bGMYC analysis based on the COI mt rRNA dataset. Colours correspond to *p*-values depicted at the right side. Clades are entered corresponding to those shown in Fig. 2.

## Data Availability

The morphological datasets supporting the conclusions of this article are included within the article and its additional files. The genetic dataset supporting the conclusions of this article is available in the GenBank repository, see Table S2 of the supporting files for accession numbers.

## Abbreviations

16S: 16S rRNA gene
ABGD: Automated Barcode Gap Discovery
AMS: Australian Museum, Sydney
AUS: Australia
bGMYC: Baysian general mixed Yule-coalescent approach
BI: Bayesian inference
BS: Bootstrap support
COI: Mitochondrial cytochrome c oxidase subunit I gene
CWR: Universität Rostock (Germany)
DNA: Deoxyribonucleic acid
EGY: Egypt
FIJ: Fiji
GMYC: General mixed Yule-coalescent approach
IND: Indonesia
IP: Indo-Polynesian Province
J: Juveniles
JP: Population found with juveniles
K2P: Kimura (K80) TS/TV 2.0 distance
MAU: Mauritius
MAD: Madagascar
MAY: Malaysia
MC^3^: Metropolis-coupled Monte Carlo Markov chain
ML: Maximum likelihood
MOZ: Mozambique
MP: Maximum parsimony
NEC: New Caledonia
NHMUK: Natural History Museum, London (United Kingdom)
OMA: Oman
PAN: Panama
PCR: Polymerase chain reaction
rRNA: Ribosomal ribonucleic acid
RS: Red Sea Province
S-Diva: Statistical dispersal-vicariance analysis
SEM: Scanning electron microscope
SMF: Senckenberg Naturmuseum, Frankfurt (Germany)
SV: RASP Value
TBR: Tree bisection and re-connection branch-swapping
THA: Thailand
V: Veliger stages
VP: Population found with veliger stages
WIO: Western Indian Ocean Province
YEM: Yemen
ZMB: Museum für Naturkunde, Berlin (Germany)
