# Supplementary material for: One species, two developmental modes: a case of geographic poecilogony in marine gastropods
Source: BMC Evol Biol. 2020 Jun 26;20:76. doi: 10.1186/s12862-020-01644-1 (PMC7318368; doi:10.1186/s12862-020-01644-1)

**Table S1.** Material examined.

| **Species** | **ID** | **Year collected** | **Sample location** | **Coordinates** |
| --- | --- | --- | --- | --- |
| *Planaxis sulcatus* | SMF 346144 |  | Eritrea, Massawa |  |
| *Planaxis sulcatus* | ZMB 117933 | 1992 | Egypt, Hurghada |  |
| *Planaxis sulcatus* | BMNH 20140857 | 1992 | Egypt, Hurghada |  |
| *Planaxis sulcatus* | ZMB 108265 | 1995 | Tanzania, Daressalam, tidal flat, N habour |  |
| *Planaxis sulcatus* | ZMB 108266 | 1995 | Tanzania, N Daressalam, Bagamoy |  |
| *Planaxis sulcatus* | AMS C.322969 | 1977 | Mozambique, Ilha Do Ibo | 12°19.810'S 40°36.330'E |
| *Planaxis sulcatus* | ZMB 117932 | 2006 | Mozambique, Mocimboa da paria |  |
| *Planaxis sulcatus* | BMNH 20140852 | 2006 | Mozambique, Mocimboa da paria |  |
| *Planaxis sulcatus* | ZMB 117936 | 2003 | Madagascar, Ste Marie Island |  |
| *Planaxis sulcatus* | BMNH 20140849 | 2003 | Madagascar, Ste Marie Island |  |
| *Planaxis sulcatus* | ZMB 117931 | 1997 | Rodaiguez, Baie Topaze |  |
| *Planaxis sulcatus* | BMNH 20140858 | 1997 | Rodaiguez, Baie Topaze |  |
| *Planaxis sulcatus* | ZMB 117937 | 2008 | Mauritius, Mont Choisy |  |
| *Planaxis sulcatus* | BMNH 20140853 | 2008 | Mauritius, Mont Choisy |  |
| *Planaxis sulcatus* | AMS C.322970 | 1977 | Mauritius, Le Chaland, semi-sheltered rocks and sand flats at hotel | 20°26.000'S 57°41.000'E |
| *Planaxis sulcatus* | SMF 346145 |  | Saudi-Arabia, Jubali, Ras-al-Bukhara |  |
| *Planaxis sulcatus* | SMF 346146 |  | Saudi-Arabia, Ras-al-Zour |  |
| *Planaxis sulcatus* | CWR 106/09 | 2009 | Yemen, al-Hudeida |  |
| *Planaxis sulcatus* | CWR 129/84 | 1984 | Yemen, Little Aden |  |
| *Planaxis sulcatus* | CWR 17/07 | 2007 | Yemen, Little Aden |  |
| *Planaxis sulcatus* | CWR 47/84 | 1984 | Yemen, Little Aden |  |
| *Planaxis sulcatus* | CWR 25/85 | 1985 | Yemen, Little Aden |  |
| *Planaxis sulcatus* | CWR 23/07 | 2007 | Yemen, Little Aden |  |
| *Planaxis sulcatus* | CWR 14/90 | 1990 | Yemen, Aden |  |
| *Planaxis sulcatus* | CWR 4/83 | 1983 | Yemen, Ras al Ara |  |
| *Planaxis sulcatus* | SMF 346147 |  | Yemen, Sokotra, south coast , Mahfirihin | 12°24.657'N 54°13.672'E |
| *Planaxis sulcatus* | SMF 346148 |  | Yemen, Sokotra, northeast coast , near Saqarah | 12°36.109'N 54°20.931'E |
| *Planaxis sulcatus* | ZMB 107847 | 1921 | Oman, E side Maqlab, Isthmus |  |
| *Planaxis sulcatus* | ZMB 107848 | 1976 | Oman, Masirah, Mothercat beach |  |
| *Planaxis sulcatus* | ZMB 107850 | 1976 | Oman, Masirah, Sea Plane Jetty |  |
| *Planaxis sulcatus* | ZMB 107849 | 1983 | Oman, Sudh, Salahah |  |
| *Planaxis sulcatus* | ZMB 117935 | 2003 | Oman, Sur |  |
| *Planaxis sulcatus* | SMF 256113 |  | Inidia, Bombay |  |
| *Planaxis sulcatus* | ZMB 117930 | 2004 | Japan, Nago, Okinawa |  |
| *Planaxis sulcatus* | BMNH 20140855 | 2004 | Japan, Nago, Okinawa |  |
| *Planaxis sulcatus* | AMS C.322976 | 1973 | China, Hongkong, Chau Wan Bay, Lamma Island | 22°14.000'N 114°7.000'E |
| *Planaxis sulcatus* | SMF 346149 |  | China, Hainan, Tien ya hai jiao |  |
| *Planaxis sulcatus* | SMF 346150 |  | China, Hainan, Xiao Donghai |  |
| *Planaxis sulcatus* | ZMB 127569a | 2013 | Thailand, North Khao Lak, Laem Pakarang | 8°44,171'N 98°13,535'E |
| *Planaxis sulcatus* | ZMB 127569b | 2013 | Thailand, North Khao Lak, Laem Pakarang | 8°44,171'N 98°13,535'E |
| *Planaxis sulcatus* | ZMB 107725 | 2010 | Thailand, Gulf of Siam, E coast of Koh Phangan, Ao Thong Nai Pan Noi | 9°46.883'N 100°3.355'E |
| *Planaxis sulcatus* | ZMB 107933 | 2010 | Thailand, Gulf of Siam, E coast of Koh Phangan, Ao Thong Nai Pan Noi | 9°46.883'N 100°3.355'E |
| *Planaxis sulcatus* | AMS C.322977 |  | Philipines, Matabungkay, Luzon Is, in small patches of mangroves | 13°57.000'N 120°37.000'E |
| *Planaxis sulcatus* | ZMB 108270 | 1993 | Malaysia, Kuantan | 3°49.000'N 103°21.000'E |
| *Planaxis sulcatus* | AMS C.322971 | 1979 | Malaysia, Mersing, E Malaya, muddy reef, near LT | 2°26.000'N 103°50.000'E |
| *Planaxis sulcatus* | ZMB 108275 | 1994 | Malaysia, Borneo, Sabah, Kota Kinabalu, Tanjung Aru, Pulau |  |
| *Planaxis sulcatus* | ZMB 108263 | 1994 | Malaysia, Sabah, Kota Kinabalu, Tanjung Aru |  |
| *Planaxis sulcatus* | ZMB 108264 | 1994 | Malaysia, Sabah, Kota Kinabalu, Tanjung Aru |  |
| *Planaxis sulcatus* | ZMB 106007 | 1999 | Indonesia, Southeast Sulawesi, Muna Island, Raha, Napapale Lagoon | 04°54.240'S 122°45.190'E |
| *Planaxis sulcatus* | ZMB 106571 | 2003 | Indonesia, Flores, NW tip of Flores, Bato Gosok, N of Labuhan Bajo, Puri Komodo Beach | 08°25.828'S 119°51.861'E |
| *Planaxis sulcatus* | ZMB 106461 | 1999 | Indonesia, Southeast Sulawesi, Raha, fortified beach in front of Hotel “Alia” | 04°50.500'S 122°43.540'E |
| *Planaxis sulcatus* | ZMB 106003 | 1999 | Indonesia, Southeast Sulawesi, Muna Island, Raha, beach nr Napapale Lagoon | 04°54.190'S 122°45.430'E |
| *Planaxis sulcatus* | ZMB 106004 | 1999 | Indonesia, Southeast Sulawesi, Muna Island, Raha, beach nr Napapale Lagoon | 04°54.190'S 122°45.430'E |
| *Planaxis sulcatus* | ZMB 106005 | 1999 | Indonesia, Southeast Sulawesi, Muna Island, Raha, fortified beach in front of Hotel “Alia” | 04°50.500'S 122°43.540'E |
| *Planaxis sulcatus* | ZMB 106006 | 1999 | Indonesia, Southeast Sulawesi, Muna Island, Raha, fortified beach in front of Hotel “Alia” | 04°50.500'S 122°43.540'E |
| *Planaxis sulcatus* | ZMB 191632 | 2005 | Indonesia, Southeast Sulawesi, Peninsula S of Malili, between Malili and Tolala, just E of Cape Pagara | 2°52.179'S 120°59.915'E |
| *Planaxis sulcatus* | AMS C.322973 | 1969 | Papua New Guinea, Louisiade Archipelago Nimoa Island, Calvados Chain & Russel Is | 11°18.000'S 153°15.000'E |
| *Planaxis sulcatus* | AMS C.322972 | 1972 | Australia, Western Australia, Coral Bay, S of Exmouth Gulf, Rocks on N side of bay | 23°7.000'S 113°46.000'E |
| *Planaxis sulcatus* | AMS C.322975 | 1970 | Australia, Northern Territory, Cape Wessel, Arnhem Land | 11°0.000'S 136°46.000'E |
| *Planaxis sulcatus* | ZMB 106372 | 2002 | Australia, Queensland, Archer Point Rock, S Cooktown | 15°36.250'S 145°19.590'E |
| *Planaxis sulcatus* | AMS C.322978 | 1963 | Australia, Queensland, Bountiful Island, Gulf of Carpentaria | 16°41.000'S 139°50.000'E |
| *Planaxis sulcatus* | ZMB 108267 | 1996 | Australia, Queensland, Cape Tribulation, Donovan Bay |  |
| *Planaxis sulcatus* | ZMB 108271 | 1996 | Australia, Queensland, Cape York, Great Barrier Reef |  |
| *Planaxis sulcatus* | AMS C.322979 | 1970 | Australia, Queensland, Halfway Island, Keppel Isles | 23°12.000'S 150°58.000'E |
| *Planaxis sulcatus* | ZMB 117934 | 2005 | Australia, Queensland, Magnetic Island, Picnic Bay |  |
| *Planaxis sulcatus* | BMNH 20140854 | 2005 | Australia, Queensland, Magnetic Island, Picnic Bay |  |
| *Planaxis sulcatus* | ZMB 108272 | 1996 | Australia, Queensland, South Mission Beach |  |
| *Planaxis sulcatus* | ZMB 108262 | 1996 | Australia, Queensland, N Cairns, Turtle Creek Point |  |
| *Planaxis sulcatus* | ZMB 107593 | 2009 | Australia, Queensland, Yule Point, south of Port Douglas |  |
| *Planaxis sulcatus* | AMS C.322974 | 1971 | New Caledonia, Ilot Maitre, in channel near Noumea | 22°20.000'S 166°24.000'E |
| *Planaxis sulcatus* | AMS C.322980 | 1971 | New Caledonia, Noumea, Magenta, High tide mark on rocks and under rocky dead coral, shore | 22°13.100'S 166°28.600'E |
| *Planaxis sulcatus* | ZMB 106376 | 2002 | Fiji, Musket Cove, Malololailai Island | 17°46.260'S 177°11.830'E |
| *Planaxis planicostatus* | ZMB 108261 | 1991 | Panama, Paitilla, Bay of Panama, Pazific |  |
| *Supplanaxis abbreviata* | ZMB 117946 | 2011 | Indonesia, Sumatra, Aceh, Ule-le |  |
| *Supplanaxis abbreviata* | ZMB 117939 | 2003 | Madagascar, Southeast Island St. Marie |  |

**Table S2.** Specimens examined for the molecular genetic analysis of this study with GenBank accession numbers.

| **Species** | **Inventory Number** | **Origin** | **16S** | **COI** | **Year collected** |
| --- | --- | --- | --- | --- | --- |
| *Planaxis sulcatus* | ZMB 106003-h1 | Indonesia |  | MT620951 | 1999 |
| *Planaxis sulcatus* | ZMB 106372-1 | Australia | MT621371 |  | 2002 |
| *Planaxis sulcatus* | ZMB 106372-2 | Australia | MT621372 |  | 2002 |
| *Planaxis sulcatus* | ZMB 106376-h2 | Fiji |  | MT620952 | 2002 |
| *Planaxis sulcatus* | ZMB 106461-1 | Indonesia | MT593028 | MT587886 | 1999 |
| *Planaxis sulcatus* | ZMB 107593-4 | Australia | MT621373 |  | 2009 |
| *Planaxis sulcatus* | ZMB 107725-4 | Thailand | MT621374 | MT620953 | 2010 |
| *Planaxis sulcatus* | ZMB 107725-6 | Thailand | MT593027 | MT587885 | 2010 |
| *Planaxis sulcatus* | ZMB 107849-4 | Oman | MT621375 |  | 1983 |
| *Planaxis sulcatus* | ZMB 107933-1 | Thailand |  | MT620954 | 2010 |
| *Planaxis sulcatus* | ZMB 107933-2 | Thailand | MT621376 | MT620955 | 2010 |
| *Planaxis sulcatus* | ZMB 107933-3 | Thailand | MT621377 | MT620956 | 2010 |
| *Planaxis sulcatus* | ZMB 108267-3 | Australia | MT621378 |  | 1996 |
| *Planaxis sulcatus* | ZMB 108275-1 | Malaysia | MT621379 |  | 1994 |
| *Planaxis sulcatus* | ZMB 108275-2 | Malaysia | MT621380 |  | 1994 |
| *Planaxis sulcatus* | ZMB 117931-1 | Mauritius | MT621381 |  | 1997 |
| *Planaxis sulcatus* | ZMB 117931-2 | Mauritius | MT593026 | MT593026 | 1997 |
| *Planaxis sulcatus* | ZMB 117932-1 | Mozambique | MT621382 |  | 2006 |
| *Planaxis sulcatus* | ZMB 117932-2 | Mozambique | MT621383 | MT620957 | 2006 |
| *Planaxis sulcatus* | ZMB 117933-1 | Egypt | MT593025 | MT587883 | 1992 |
| *Planaxis sulcatus* | ZMB 117933-2 | Egypt | MT621384 | MT620958 | 1992 |
| *Planaxis sulcatus* | ZMB 117936-1 | Madagascar | MT621385 | MT620959 | 2003 |
| *Planaxis sulcatus* | ZMB 117937-1 | Mauritius | MT621386 |  | 2008 |
| *Planaxis sulcatus* | ZMB 127569a-1 | Thailand | MT621387 | MT620960 | 2013 |
| *Planaxis sulcatus* | ZMB 127569b-1 | Thailand | MT621388 | MT620961 | 2013 |
| *Planaxis sulcatus* | ZMB 127569b-2 | Thailand | MT621389 | MT620962 | 2013 |
| *Planaxis sulcatus* | ZMB 191632-9 | Indonesia | MT621390 |  | 2005 |
| *Planaxis sulcatus* | AMS C. 322974-h2 | New Caledonia | MT621367 |  | 1971 |
| *Planaxis sulcatus* | CWR 106/09-2 | Yemen | MT621368 |  | 2009 |
| *Planaxis sulcatus* | CWR 17/07-1 | Yemen | MT621369 |  | 2007 |
| *Planaxis sulcatus* | CWR 17/07-2 | Yemen | MT621370 |  | 2007 |
| *Planaxis planicostatus* | ZMB 108261-h1 | Panama | MT621366 |  | 1991 |
| *Supplanaxis abbreviata* | ZMB 117939-1 | Madagascar | MT593021 | MT587879 | 2003 |
| *Supplanaxis abbreviata* | ZMB 117946-1 | Indonesia | MT593020 | MT587878 | 2011 |

**Table S3.** Results of brood pouch content analysis of *Planaxis sulcatus*. If present, content was counted according to five predefined size classes.

| ID | Country of sample origin | Total amount of offspring | Eggs/Early larvae | late larvae | Juvenile < 0.5 mm | Juvenile 0.6–1.0 mm | Juvenile 1.1–1.5 mm |
| --- | --- | --- | --- | --- | --- | --- | --- |
| AMS 322972-1 | Australia |  |  |  |  |  |  |
| AMS 322972-2 | Australia |  |  |  |  |  |  |
| AMS 322972-3 | Australia |  |  |  |  |  |  |
| AMS 322972-4 | Australia |  |  |  |  |  |  |
| AMS 322972-5 | Australia |  |  |  |  |  |  |
| AMS 322972-6 | Australia |  |  |  |  |  |  |
| AMS 322975-1 | Australia |  |  |  |  |  |  |
| AMS 322975-2 | Australia |  |  |  |  |  |  |
| AMS 322975-3 | Australia |  |  |  |  |  |  |
| AMS 322975-4 | Australia | 3000 | 3000 | 0 | 0 | 0 | 0 |
| AMS 322979-1 | Australia |  |  |  |  |  |  |
| AMS 322979-2 | Australia |  |  |  |  |  |  |
| AMS 322979-3 | Australia |  |  |  |  |  |  |
| AMS 322979-4 | Australia |  |  |  |  |  |  |
| AMS 322979-5 | Australia |  |  |  |  |  |  |
| ZMB 106372-1 | Australia |  |  |  |  |  |  |
| ZMB 106372-2 | Australia |  |  |  |  |  |  |
| ZMB 106372-3 | Australia |  |  |  |  |  |  |
| ZMB 106372-4 | Australia |  |  |  |  |  |  |
| ZMB 106372-5 | Australia |  |  |  |  |  |  |
| ZMB 106372-6 | Australia |  |  |  |  |  |  |
| ZMB 106372-7 | Australia |  |  |  |  |  |  |
| ZMB 107593-1 | Australia |  |  |  |  |  |  |
| ZMB 107593-2 | Australia |  |  |  |  |  |  |
| ZMB 107593-3 | Australia |  |  |  |  |  |  |
| ZMB 107593-4 | Australia |  |  |  |  |  |  |
| ZMB 107593-5 | Australia |  |  |  |  |  |  |
| ZMB 107593-6 | Australia | 1700 | 1700 | 0 | 0 | 0 | 0 |
| ZMB 108262-1 | Australia |  |  |  |  |  |  |
| ZMB 108262-2 | Australia |  |  |  |  |  |  |
| ZMB 108262-3 | Australia |  |  |  |  |  |  |
| ZMB 108262-4 | Australia |  |  |  |  |  |  |
| ZMB 108262-5 | Australia |  |  |  |  |  |  |
| ZMB 108267-1 | Australia |  |  |  |  |  |  |
| ZMB 108267-2 | Australia |  |  |  |  |  |  |
| ZMB 108267-3 | Australia |  |  |  |  |  |  |
| ZMB 108267-4 | Australia |  |  |  |  |  |  |
| ZMB 108271-5 | Australia |  |  |  |  |  |  |
| ZMB 108271-6 | Australia |  |  |  |  |  |  |
| ZMB 108271-7 | Australia |  |  |  |  |  |  |
| ZMB 108271-8 | Australia |  |  |  |  |  |  |
| ZMB 108271-9 | Australia |  |  |  |  |  |  |
| ZMB 108272-1 | Australia |  |  |  |  |  |  |
| ZMB 108272-2 | Australia |  |  |  |  |  |  |
| ZMB 108272-3 | Australia |  |  |  |  |  |  |
| ZMB 108272-4 | Australia |  |  |  |  |  |  |
| ZMB 107593-1 | Australia |  |  |  |  |  |  |
| ZMB 107593-2 | Australia |  |  |  |  |  |  |
| ZMB 107593-3 | Australia |  |  |  |  |  |  |
| ZMB 107593-4 | Australia |  |  |  |  |  |  |
| ZMB 107593-5 | Australia |  |  |  |  |  |  |
| ZMB 107593-6 | Australia |  |  |  |  |  |  |
| ZMB 107593-7 | Australia |  |  |  |  |  |  |
| ZMB 107593-8 | Australia |  |  |  |  |  |  |
| ZMB 107593-9 | Australia |  |  |  |  |  |  |
| ZMB 108271-10 | Australia |  |  |  |  |  |  |
| ZMB 108271-11 | Australia |  |  |  |  |  |  |
| SMF 346149-1 | China |  |  |  |  |  |  |
| SMF 346149-2 | China |  |  |  |  |  |  |
| SMF 346149-3 | China |  |  |  |  |  |  |
| SMF 346150-1 | China |  |  |  |  |  |  |
| SMF 346150-2 | China |  |  |  |  |  |  |
| SMF 346150-3 | China |  |  |  |  |  |  |
| SMF 346150-4 | China |  |  |  |  |  |  |
| SMF 346150-5 | China |  |  |  |  |  |  |
| SMF 346150-6 | China |  |  |  |  |  |  |
| SMF 346144-1 | Eritrea |  |  |  |  |  |  |
| SMF 346144-2 | Eritrea |  |  |  |  |  |  |
| SMF 346144-3 | Eritrea | 28 | 0 | 0 | 0 | 28 | 0 |
| SMF 346144-4 | Eritrea | 115 | 0 | 35 | 0 | 30 | 50 |
| SMF 346144-5 | Eritrea | 330 | 0 | 330 | 0 | 0 | 0 |
| SMF 346144-6 | Eritrea |  |  |  |  |  |  |
| ZMB 117933-1 | Egypt |  |  |  |  |  |  |
| ZMB 117933-2 | Egypt |  |  |  |  |  |  |
| ZMB 106376-7 | Fiji |  |  |  |  |  |  |
| ZMB 106376-8 | Fiji |  |  |  |  |  |  |
| ZMB 106376-9 | Fiji |  |  |  |  |  |  |
| ZMB 106376-10 | Fiji |  |  |  |  |  |  |
| ZMB 106376-11 | Fiji |  |  |  |  |  |  |
| ZMB 106376-12 | Fiji |  |  |  |  |  |  |
| ZMB 106376-13 | Fiji | 1060 | 0 | 1060 | 0 | 0 | 0 |
| ZMB 106376-14 | Fiji |  |  |  |  |  |  |
| ZMB 106376-h1 | Fiji |  |  |  |  |  |  |
| ZMB 106376-h2 | Fiji |  |  |  |  |  |  |
| SMF 256113-1 | India | 4900 | 0 | 4900 | 0 | 0 | 0 |
| SMF 256113-2 | India | 3350 | 0 | 3350 | 0 | 0 | 0 |
| SMF 256114-1 | India |  |  |  |  |  |  |
| SMF 256114-2 | India |  |  |  |  |  |  |
| SMF 256114-3 | India |  |  |  |  |  |  |
| SMF 256114-4 | India |  |  |  |  |  |  |
| SMF 256114-5 | India |  |  |  |  |  |  |
| ZMB 106003-1 | Indonesia |  |  |  |  |  |  |
| ZMB 106003-2 | Indonesia |  |  |  |  |  |  |
| ZMB 106003-3 | Indonesia |  |  |  |  |  |  |
| ZMB 106003-4 | Indonesia |  |  |  |  |  |  |
| ZMB 106003-5 | Indonesia |  |  |  |  |  |  |
| ZMB 106003-6 | Indonesia |  |  |  |  |  |  |
| ZMB 106003-7 | Indonesia |  |  |  |  |  |  |
| ZMB 106003-8 | Indonesia |  |  |  |  |  |  |
| ZMB 106003-9 | Indonesia |  |  |  |  |  |  |
| ZMB 106003-10 | Indonesia |  |  |  |  |  |  |
| ZMB 106003-11 | Indonesia |  |  |  |  |  |  |
| ZMB 106003-12 | Indonesia |  |  |  |  |  |  |
| ZMB 106003-13 | Indonesia |  |  |  |  |  |  |
| ZMB 106003-h1 | Indonesia |  |  |  |  |  |  |
| ZMB 106004-1 | Indonesia |  |  |  |  |  |  |
| ZMB 106004-2 | Indonesia |  |  |  |  |  |  |
| ZMB 106004-3 | Indonesia |  |  |  |  |  |  |
| ZMB 106004-4 | Indonesia |  |  |  |  |  |  |
| ZMB 106004-5 | Indonesia |  |  |  |  |  |  |
| ZMB 106004-6 | Indonesia |  |  |  |  |  |  |
| ZMB 106004-7 | Indonesia | 1400 | 1400 | 0 | 0 | 0 | 0 |
| ZMB 106004-8 | Indonesia |  |  |  |  |  |  |
| ZMB 106004-9 | Indonesia |  |  |  |  |  |  |
| ZMB 106004-10 | Indonesia |  |  |  |  |  |  |
| ZMB 106004-11 | Indonesia |  |  |  |  |  |  |
| ZMB 106004-12 | Indonesia |  |  |  |  |  |  |
| ZMB 106005-1 | Indonesia | 2900 | 2900 | 0 | 0 | 0 | 0 |
| ZMB 106005-2 | Indonesia |  |  |  |  |  |  |
| ZMB 106005-3 | Indonesia |  |  |  |  |  |  |
| ZMB 106005-4 | Indonesia |  |  |  |  |  |  |
| ZMB 106005-5 | Indonesia |  |  |  |  |  |  |
| ZMB 106005-6 | Indonesia |  |  |  |  |  |  |
| ZMB 106005-7 | Indonesia |  |  |  |  |  |  |
| ZMB 106005-8 | Indonesia |  |  |  |  |  |  |
| ZMB 106005-9 | Indonesia |  |  |  |  |  |  |
| ZMB 106005-10 | Indonesia |  |  |  |  |  |  |
| ZMB 106005-11 | Indonesia |  |  |  |  |  |  |
| ZMB 106005-12 | Indonesia |  |  |  |  |  |  |
| ZMB 106006-1 | Indonesia |  |  |  |  |  |  |
| ZMB 106006-2 | Indonesia |  |  |  |  |  |  |
| ZMB 106006-3 | Indonesia |  |  |  |  |  |  |
| ZMB 106006-4 | Indonesia |  |  |  |  |  |  |
| ZMB 106006-5 | Indonesia |  |  |  |  |  |  |
| ZMB 106006-6 | Indonesia |  |  |  |  |  |  |
| ZMB 106006-7 | Indonesia |  |  |  |  |  |  |
| ZMB 106006-8 | Indonesia |  |  |  |  |  |  |
| ZMB 106006-9 | Indonesia |  |  |  |  |  |  |
| ZMB 106007-1 | Indonesia |  |  |  |  |  |  |
| ZMB 106007-2 | Indonesia |  |  |  |  |  |  |
| ZMB 106007-3 | Indonesia |  |  |  |  |  |  |
| ZMB 106007-4 | Indonesia | 1350 | 1350 | 0 | 0 | 0 | 0 |
| ZMB 106007-5 | Indonesia |  |  |  |  |  |  |
| ZMB 106007-6 | Indonesia | 1550 | 1550 | 0 | 0 | 0 | 0 |
| ZMB 106007-8 | Indonesia |  |  |  |  |  |  |
| ZMB 106007-9 | Indonesia |  |  |  |  |  |  |
| ZMB 106007-10 | Indonesia |  |  |  |  |  |  |
| ZMB 106461-1 | Indonesia |  |  |  |  |  |  |
| ZMB 106571-10 | Indonesia |  |  |  |  |  |  |
| ZMB 106571-11 | Indonesia |  |  |  |  |  |  |
| ZMB 106571-12 | Indonesia |  |  |  |  |  |  |
| ZMB 106571-12 | Indonesia |  |  |  |  |  |  |
| ZMB 106571-13 | Indonesia |  |  |  |  |  |  |
| ZMB 106571-14 | Indonesia |  |  |  |  |  |  |
| ZMB 191632-1 | Indonesia |  |  |  |  |  |  |
| ZMB 191632-2 | Indonesia |  |  |  |  |  |  |
| ZMB 191632-3 | Indonesia |  |  |  |  |  |  |
| ZMB 191632-4 | Indonesia |  |  |  |  |  |  |
| ZMB 191632-5 | Indonesia |  |  |  |  |  |  |
| ZMB 191632-6 | Indonesia |  |  |  |  |  |  |
| ZMB 191632-7 | Indonesia | 2100 | 0 | 2100 | 0 | 0 | 0 |
| ZMB 191632-8 | Indonesia |  |  |  |  |  |  |
| ZMB 191632-9 | Indonesia |  |  |  |  |  |  |
| ZMB 191632-10 | Indonesia |  |  |  |  |  |  |
| ZMB 191632-11 | Indonesia |  |  |  |  |  |  |
| ZMB 117936-1 | Madagascar |  |  |  |  |  |  |
| ZMB 108263-1 | Malaysia |  |  |  |  |  |  |
| ZMB 108263-2 | Malaysia |  |  |  |  |  |  |
| ZMB 108263-3 | Malaysia |  |  |  |  |  |  |
| ZMB 108263-4 | Malaysia |  |  |  |  |  |  |
| ZMB 108263-5 | Malaysia |  |  |  |  |  |  |
| ZMB 108263-6 | Malaysia |  |  |  |  |  |  |
| ZMB 108264-1 | Malaysia |  |  |  |  |  |  |
| ZMB 108264-2 | Malaysia |  |  |  |  |  |  |
| ZMB 108264-3 | Malaysia |  |  |  |  |  |  |
| ZMB 108270-1 | Malaysia |  |  |  |  |  |  |
| ZMB 108270-2 | Malaysia |  |  |  |  |  |  |
| ZMB 108270-3 | Malaysia |  |  |  |  |  |  |
| ZMB 108270-4 | Malaysia |  |  |  |  |  |  |
| ZMB 108275-1 | Malaysia |  |  |  |  |  |  |
| ZMB 108275-2 | Malaysia |  |  |  |  |  |  |
| ZMB 108275-3 | Malaysia |  |  |  |  |  |  |
| ZMB 108275-4 | Malaysia |  |  |  |  |  |  |
| AMS 322971-1 | Malaysia |  |  |  |  |  |  |
| AMS 322971-2 | Malaysia |  |  |  |  |  |  |
| AMS 322971-3 | Malaysia |  |  |  |  |  |  |
| AMS 322970-1 | Mauritius | 1200 | 1200 | 0 | 0 | 0 | 0 |
| AMS 322970-2 | Mauritius |  |  |  |  |  |  |
| AMS 322970-3 | Mauritius |  |  |  |  |  |  |
| ZMB 117931-1 | Mauritius |  |  |  |  |  |  |
| ZMB 117931-2 | Mauritius |  |  |  |  |  |  |
| ZMB 117937-1 | Mauritius |  |  |  |  |  |  |
| AMS 322969-1 | Mozambique | 1300 | 1300 | 0 | 0 | 0 | 0 |
| AMS 322969-2 | Mozambique | 1900 | 1900 | 0 | 0 | 0 | 0 |
| AMS 322969-3 | Mozambique |  |  |  |  |  |  |
| AMS 322969-4 | Mozambique |  |  |  |  |  |  |
| AMS 322969-5 | Mozambique |  |  |  |  |  |  |
| AMS 322969-6 | Mozambique |  |  |  |  |  |  |
| AMS 322969-7 | Mozambique | 2511 | 2500 | 11 | 0 | 0 | 0 |
| AMS 322969-8 | Mozambique | 32 | 0 | 0 | 0 | 32 | 0 |
| AMS 322969-9 | Mozambique |  |  |  |  |  |  |
| AMS 322969-10 | Mozambique |  |  |  |  |  |  |
| AMS 322969-11 | Mozambique |  |  |  |  |  |  |
| ZMB 117932-1 | Mozambique |  |  |  |  |  |  |
| ZMB 117932-2 | Mozambique |  |  |  |  |  |  |
| AMS 322974-1 | New Caledonia |  |  |  |  |  |  |
| AMS 322974-2 | New Caledonia |  |  |  |  |  |  |
| AMS 322974-3 | New Caledonia |  |  |  |  |  |  |
| AMS 322974-4 | New Caledonia |  |  |  |  |  |  |
| AMS 322974-5 | New Caledonia |  |  |  |  |  |  |
| AMS 322974-6 | New Caledonia |  |  |  |  |  |  |
| AMS 322974-7 | New Caledonia |  |  |  |  |  |  |
| AMS 322974-8 | New Caledonia |  |  |  |  |  |  |
| AMS 322974-9 | New Caledonia |  |  |  |  |  |  |
| AMS 322974-10 | New Caledonia | 688 | 273 | 415 | 0 | 0 | 0 |
| AMS 322974-11 | New Caledonia |  |  |  |  |  |  |
| AMS 322974-12 | New Caledonia |  |  |  |  |  |  |
| AMS 322974-h1 | New Caledonia |  |  |  |  |  |  |
| AMS 322974-h2 | New Caledonia |  |  |  |  |  |  |
| AMS 322980-1 | New Caledonia |  |  |  |  |  |  |
| AMS 322980-2 | New Caledonia |  |  |  |  |  |  |
| AMS 322980-3 | New Caledonia |  |  |  |  |  |  |
| AMS 322980-4 | New Caledonia | 1700 | 0 | 1700 | 0 | 0 | 0 |
| AMS 322980-5 | New Caledonia | 2100 | 0 | 2100 | 0 | 0 | 0 |
| AMS 322980-6 | New Caledonia |  |  |  |  |  |  |
| ZMB 107847-1 | Oman |  |  |  |  |  |  |
| ZMB 107847-2 | Oman |  |  |  |  |  |  |
| ZMB 107847-3 | Oman |  |  |  |  |  |  |
| ZMB 107847-4 | Oman |  |  |  |  |  |  |
| ZMB 107847-5 | Oman |  |  |  |  |  |  |
| ZMB 107848-1 | Oman |  |  |  |  |  |  |
| ZMB 107848-2 | Oman |  |  |  |  |  |  |
| ZMB 107848-3 | Oman | 170 | 0 | 0 | 0 | 170 | 0 |
| ZMB 107848-4 | Oman |  |  |  |  |  |  |
| ZMB 107848-5 | Oman | 98 | 0 | 0 | 0 | 98 | 0 |
| ZMB 107849-1 | Oman |  |  |  |  |  |  |
| ZMB 107849-2 | Oman |  |  |  |  |  |  |
| ZMB 107849-3 | Oman |  |  |  |  |  |  |
| ZMB 107849-4 | Oman |  |  |  |  |  |  |
| ZMB 107849-5 | Oman | 292 | 0 | 0 | 0 | 292 | 0 |
| ZMB 107850-1 | Oman |  |  |  |  |  |  |
| ZMB 107850-2 | Oman |  |  |  |  |  |  |
| ZMB 107850-3 | Oman | 562 | 0 | 0 | 0 | 562 | 0 |
| ZMB 107850-4 | Oman |  |  |  |  |  |  |
| ZMB 107850-5 | Oman | 150 | 0 | 0 | 0 | 150 | 0 |
| ZMB 107850-6 | Oman | 107 | 0 | 0 | 0 | 107 | 0 |
| ZMB 107850-7 | Oman | 304 | 0 | 0 | 0 | 304 | 0 |
| ZMB 107850-8 | Oman |  |  |  |  |  |  |
| ZMB 107850-9 | Oman |  |  |  |  |  |  |
| ZMB 107850-10 | Oman | 137 | 0 | 0 | 0 | 137 | 0 |
| ZMB 107849-6 | Oman |  |  |  |  |  |  |
| AMS 322973-1 | Papua New Guinea | 1550 | 1550 | 0 | 0 | 0 | 0 |
| AMS 322973-2 | Papua New Guinea | 30 | 30 | 0 | 0 | 0 | 0 |
| AMS 322973-3 | Papua New Guinea |  |  |  |  |  |  |
| AMS 322973-4 | Papua New Guinea |  |  |  |  |  |  |
| AMS 322973-5 | Papua New Guinea |  |  |  |  |  |  |
| AMS 322973-6 | Papua New Guinea |  |  |  |  |  |  |
| AMS 322973-7 | Papua New Guinea |  |  |  |  |  |  |
| AMS 322973-8 | Papua New Guinea |  |  |  |  |  |  |
| AMS 322973-9 | Papua New Guinea |  |  |  |  |  |  |
| SMF 346145-1 | Saudi-Arabia | 380 | 0 | 0 | 380 | 0 | 0 |
| SMF 346145-2 | Saudi-Arabia | 73 | 73 | 0 | 0 | 0 | 0 |
| SMF 346145-3 | Saudi-Arabia | 94 | 94 | 0 | 0 | 0 | 0 |
| SMF 346145-4 | Saudi-Arabia | 70 | 70 | 0 | 0 | 0 | 0 |
| SMF 346145-5 | Saudi-Arabia | 268 | 0 | 0 | 268 | 0 | 0 |
| SMF 346145-6 | Saudi-Arabia | 26 | 0 | 0 | 1 | 25 | 0 |
| SMF 346146-1 | Saudi-Arabia | 110 | 0 | 0 | 110 | 0 | 0 |
| SMF 346146-2 | Saudi-Arabia | 350 | 0 | 0 | 350 | 0 | 0 |
| SMF 346146-3 | Saudi-Arabia | 163 | 0 | 0 | 163 | 0 | 0 |
| SMF 346146-4 | Saudi-Arabia | 230 | 0 | 0 | 230 | 0 | 0 |
| SMF 346146-5 | Saudi-Arabia | 220 | 0 | 0 | 220 | 0 | 0 |
| ZMB 108266-5 | Tanzania |  |  |  |  |  |  |
| ZMB 108265-14 | Tanzania |  |  |  |  |  |  |
| ZMB 108265-15 | Tanzania | 6980 | 0 | 0 | 6980 | 0 | 0 |
| ZMB 108265-16 | Tanzania |  |  |  |  |  |  |
| ZMB 108265-17 | Tanzania |  |  |  |  |  |  |
| ZMB 108265-18 | Tanzania |  |  |  |  |  |  |
| ZMB 108265-19 | Tanzania |  |  |  |  |  |  |
| ZMB 108265-20 | Tanzania |  |  |  |  |  |  |
| ZMB 107933-1 | Thailand |  |  |  |  |  |  |
| ZMB 107933-2 | Thailand |  |  |  |  |  |  |
| ZMB 107933-3 | Thailand |  |  |  |  |  |  |
| ZMB 107725-1 | Thailand |  |  |  |  |  |  |
| ZMB 107725-2 | Thailand |  |  |  |  |  |  |
| ZMB 107725-3 | Thailand |  |  |  |  |  |  |
| ZMB 107725-4 | Thailand |  |  |  |  |  |  |
| ZMB 107725-5 | Thailand |  |  |  |  |  |  |
| ZMB 107725-6 | Thailand |  |  |  |  |  |  |
| ZMB 127569a-1 | Thailand |  |  |  |  |  |  |
| ZMB 127569b-1 | Thailand |  |  |  |  |  |  |
| ZMB 127569b-2 | Thailand |  |  |  |  |  |  |
| ZMB 127569b-3 | Thailand |  |  |  |  |  |  |
| ZMB 127569b-4 | Thailand |  |  |  |  |  |  |
| ZMB 127569b-5 | Thailand |  |  |  |  |  |  |
| ZMB 127569b-6 | Thailand |  |  |  |  |  |  |
| ZMB 127569b-7 | Thailand |  |  |  |  |  |  |
| ZMB 127569b-8 | Thailand |  |  |  |  |  |  |
| ZMB 127569b-9 | Thailand |  |  |  |  |  |  |
| ZMB 127569b-10 | Thailand |  |  |  |  |  |  |
| CWR 129/84-1 | Yemen |  |  |  |  |  |  |
| CWR 129/84-2 | Yemen | 2300 | 0 | 2300 | 0 | 0 | 0 |
| CWR 129/84-3 | Yemen |  |  |  |  |  |  |
| CWR 129/84-4 | Yemen | 95 | 0 | 15 | 80 | 0 | 0 |
| CWR 129/84-5 | Yemen |  |  |  |  |  |  |
| CWR 129/84-6 | Yemen |  |  |  |  |  |  |
| CWR 129/84-7 | Yemen | 2680 | 2680 | 0 | 0 | 0 | 0 |
| CWR 129/84-8 | Yemen |  |  |  |  |  |  |
| CWR 129/84-9 | Yemen |  |  |  |  |  |  |
| CWR 129/84-10 | Yemen | 10500 | 10500 | 0 | 0 | 0 | 0 |
| CWR 106/09-1 | Yemen | 20 | 0 | 20 | 0 | 0 | 0 |
| CWR 106/09-2 | Yemen | 1 | 0 | 1 | 0 | 0 | 0 |
| CWR 106/09-3 | Yemen |  |  |  |  |  |  |
| CWR 106/09-4 | Yemen |  |  |  |  |  |  |
| CWR 106/09-5 | Yemen |  |  |  |  |  |  |
| CWR 106/09-6 | Yemen |  |  |  |  |  |  |
| CWR 106/09-7 | Yemen |  |  |  |  |  |  |
| CWR 17/07-1 | Yemen |  |  |  |  |  |  |
| CWR 17/07-2 | Yemen |  |  |  |  |  |  |
| CWR 17/07-3 | Yemen |  |  |  |  |  |  |
| CWR 17/07-4 | Yemen |  |  |  |  |  |  |
| CWR 17/07-5 | Yemen |  |  |  |  |  |  |
| CWR 47/84-1 | Yemen |  |  |  |  |  |  |
| CWR 47/84-2 | Yemen |  |  |  |  |  |  |
| CWR 47/84-3 | Yemen |  |  |  |  |  |  |
| CWR 47/84-4 | Yemen |  |  |  |  |  |  |
| CWR 47/84-5 | Yemen | 318 | 0 | 0 | 0 | 318 | 0 |
| CWR 47/84-6 | Yemen |  |  |  |  |  |  |
| CWR 47/84-7 | Yemen |  |  |  |  |  |  |
| CWR 47/84-8 | Yemen | 180 | 0 | 0 | 180 | 0 | 0 |
| CWR 47/84-9 | Yemen |  |  |  |  |  |  |
| CWR 47/84-10 | Yemen | 4 | 0 | 0 | 0 | 4 | 0 |
| CWR 14/90-1 | Yemen | 3100 | 2300 | 800 | 0 | 0 | 0 |
| CWR 14/90-2 | Yemen |  |  |  |  |  |  |
| CWR 14/90-3 | Yemen |  |  |  |  |  |  |
| CWR 14/90-4 | Yemen |  |  |  |  |  |  |
| CWR 14/90-5 | Yemen |  |  |  |  |  |  |
| CWR 14/90-6 | Yemen |  |  |  |  |  |  |
| CWR 4/83-1 | Yemen |  |  |  |  |  |  |
| CWR 4/83-2 | Yemen |  |  |  |  |  |  |
| CWR 4/83-3 | Yemen |  |  |  |  |  |  |
| CWR 4/83-4 | Yemen |  |  |  |  |  |  |
| CWR 4/83-5 | Yemen |  |  |  |  |  |  |
| CWR 25/85-1 | Yemen |  |  |  |  |  |  |
| CWR 25/85-2 | Yemen |  |  |  |  |  |  |
| CWR 25/85-3 | Yemen |  |  |  |  |  |  |
| CWR 25/85-4 | Yemen |  |  |  |  |  |  |
| CWR 25/85-5 | Yemen |  |  |  |  |  |  |
| CWR 25/85-6 | Yemen |  |  |  |  |  |  |
| CWR 25/85-7 | Yemen |  |  |  |  |  |  |
| CWR 25/85-8 | Yemen |  |  |  |  |  |  |
| CWR 25/85-9 | Yemen |  |  |  |  |  |  |
| CWR 25/85-10 | Yemen |  |  |  |  |  |  |
| SMF 346147-1 | Yemen | 212 | 0 | 0 | 212 | 0 | 0 |
| SMF 346147-2 | Yemen | 263 | 0 | 0 | 263 | 0 | 0 |
| SMF 346147-3 | Yemen | 249 | 0 | 0 | 249 | 0 | 0 |
| SMF 346147-4 | Yemen | 275 | 0 | 0 | 273 | 2 | 0 |
| SMF 346147-5 | Yemen | 270 | 0 | 0 | 270 | 0 | 0 |
| SMF 346147-6 | Yemen | 270 | 0 | 0 | 270 | 0 | 0 |
| SMF 346148-1 | Yemen | 392 | 0 | 0 | 392 | 0 | 0 |
| SMF 346148-2 | Yemen | 180 | 0 | 0 | 180 | 0 | 0 |
| SMF 346148-3 | Yemen | 2400 | 0 | 2400 | 0 | 0 | 0 |
| SMF 346148-4 | Yemen |  |  |  |  |  |  |
| SMF 346148-5 | Yemen | 28 | 0 | 28 | 0 | 0 | 0 |
| SMF 346148-6 | Yemen | 63 | 0 | 0 | 63 | 0 | 0 |

**Table S4.** Results of ABGD analysis for the COI mt rRNA dataset using the K2P model to calculate pairwise distances. P is the prior maximum intraspecific divergence.

| **Partition No.** | **Groups** | ***P*-Value** |
| --- | --- | --- |
| 1 | 3 | 0.003100 |
| 2 | 3 | 0.004131 |
| 3 | 3 | 0.005506 |
| 4 | 3 | 0.007337 |
| 5 | 3 | 0.009778 |
| 6 | 2 | 0.013031 |
| 7 | 2 | 0.017365 |
| 8 | 2 | 0.023142 |
| 9 | 1 | 0.030800 |

**Figure S1.** Pobability matrix of the bGMYC analysis based on the COI mt rRNA dataset. Colours correspond to p-values depicted at the right side. Clades are entered corresponding to those shown in Figure 2.


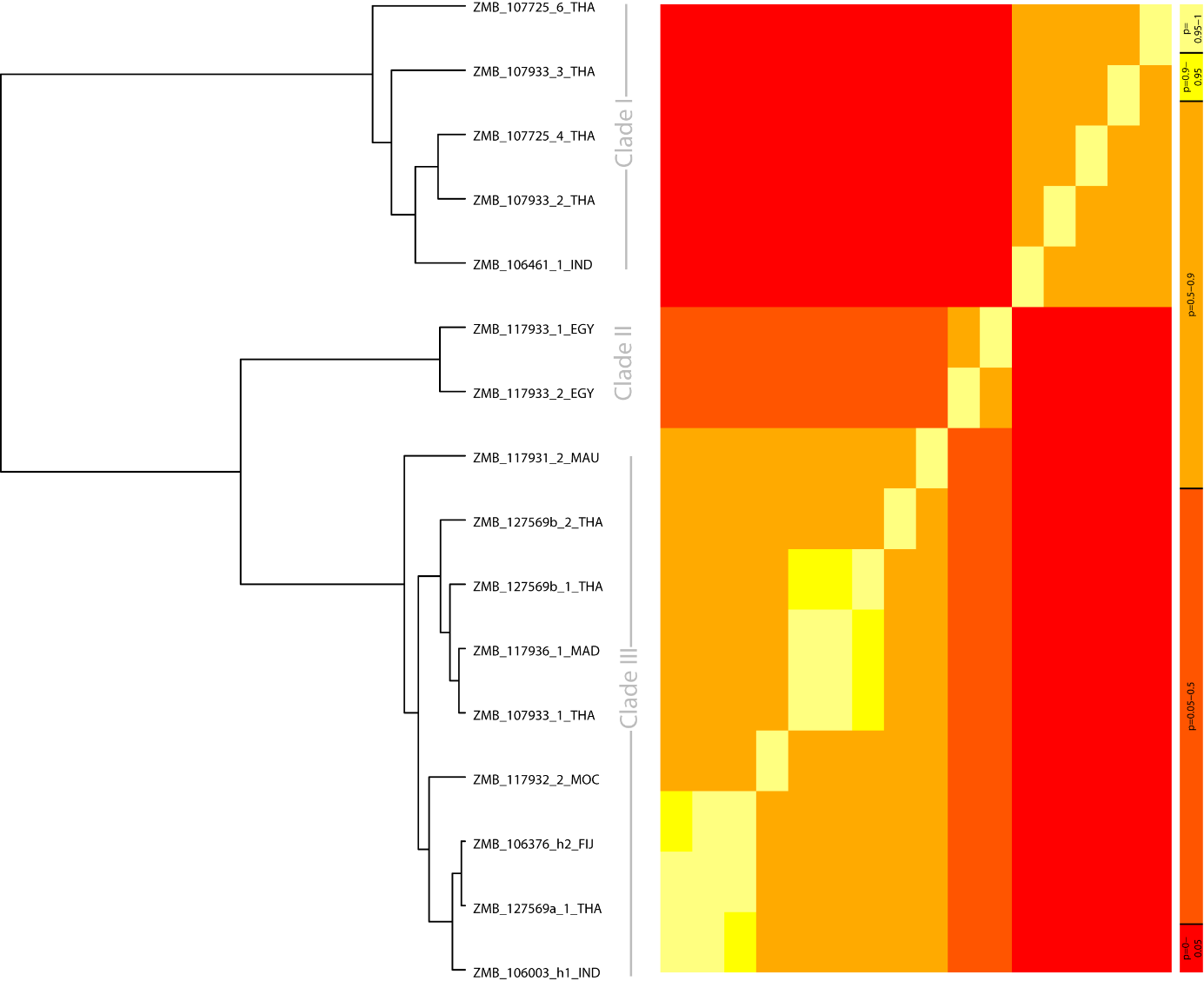

Supplement: Supplementary file 1 — Additional file 1 Table S1 Material examined. Table S2 Specimens examined for the molecular genetic analysis of this study with GenBank accession and inventory numbers. Table S3 Results of brood pouch content analysis of Planaxis sulcatus. If present, content was counted according to five predefined size classes. Table S4 Results of ABGD analysis for the COI mt rRNA dataset using the K2P model to calculate pairwise distances. P is the prior maximum intraspecific divergence. Fig. S1 Pobability matrix of the bGMYC analysis based on the COI mt rRNA dataset. Colours correspond to p-values depicted at the right side. Clades are entered corresponding to those shown in Fig. 2. [file 12862_2020_1644_MOESM1_ESM.docx]
